# Supplementary material for: Health-related quality of life in Brazilian patients with cutaneous leishmaniasis using EQ-5D
Source: PLoS One. 2025 May 23;20(5):e0324788. doi: 10.1371/journal.pone.0324788 (PMC12101690; doi:10.1371/journal.pone.0324788)
Supplement: S2 Appendix — (DOCX) [file pone.0324788.s002.docx]

The questions we will ask are related to your health. As we have explained, the objective of this study is to investigate the quality of life of patients diagnosed with leishmaniasis. For us, it is very important to know how you are feeling and how you have been lately. There are no right or wrong answers. And if you have any questions, I am here to help.

1. Overall, how would you rate your health state before the symptoms of leishmaniasis?

❑1. Very good

❑2. Good

❑3. Average

❑4. Bad

❑5. Very bad

1. Now, I would like you to tell me about your capacity to walk around the house or in the street. Tell me which of the statements below best describes your **mobility before the symptoms of leishmaniasis?**

❑ 1. I have no problems in walking about

❑ 2. I have some problems in walking about

❑ 3. I am confined to bed

1. What about your **self-care**, such as taking a shower, getting dressed, eating? Which of the states below best describes your ability to perform these activities **before the symptoms of leishmaniasis?**

❑ 1. I have no problems with self-care

❑ 2. I have some problems washing or dressing myself

❑ 3. I am unable to wash or dress myself

1. Another important health aspect is the ability to perform daily tasks, such as working, studying, taking care of the house, meeting friends and neighbors, going to church. Tell me, which of the statements below best describes your ability to perform such **usual activities before the symptoms of leishmaniasis?**

❑ 1. I have no problems with performing my usual activities

❑ 2. I have some problems with performing my usual activities

❑ 3. I am unable to perform my usual activities

1. Some diseases or health conditions can cause **pain or disconfort,** such as a headache, a stomachage, back pain, any kind of pain... Tell me which of the statements below best describes your health **before the symptoms of leishmaniasis?**

❑ 1. I have no pain or disconfort

❑ 2. I have moderate pain or disconfort

❑ 3. I have pain or disconfort

1. As for **anxiety and depression**, there is to say, fear, panic, anguish, persistent worrying, trouble sleeping, deep sadness, discouragement, lack of energy... Which alternative bests describes how you have been feeling **before the symptoms of leishmaniasis?**

❑ 1. I am not anxious or depressed

❑ 2. I am moderately anxious or depressed

❑ 3. I am extremely anxious or depressed

Best imaginable health state

90

80

70

60

50

40

30

20

10

100

0

Now, I would like you to show your health state using this ruler. In it, 100 is the best health state and 0 is the worst. Please, use a line to mark the point that represents your health state before the symptoms of leishmaniasis.

Worst imaginable health state
