# Supplementary material for: Health-related quality of life in Brazilian patients with cutaneous leishmaniasis using EQ-5D
Source: PLoS One. 2025 May 23;20(5):e0324788. doi: 10.1371/journal.pone.0324788 (PMC12101690; doi:10.1371/journal.pone.0324788)
Supplement: S3 Appendix — (DOCX) [file pone.0324788.s003.docx]

*Reminder to the interviewer: this part of the tool should only be applied in the first interview.*

I will now ask some questions about household items for an economic assessment. All the electronics items I will mention should be working, including the ones that are put away. For the items that are not working, make sure you only include them in case you plan on either fixing or replacing them in the next six months.

**INSTRUCTION**: All the question items should be asked by the interviewer and answered by the interviewee.

**Shall we begin? In your household, there is/are: (**READ EACH ITEM)

|  | | **ITEM NUMBER** | | | |
| --- | --- | --- | --- | --- | --- |
| **COMFORT ITEMS** | **NO** | **1** | **2** | **3** | **4+** |
| 1. Number of private leisure vehicles |  |  |  |  |  |
| 1. Number of monthly emplyoees, considering only those who work at least five days a week |  |  |  |  |  |
| 1. Number of washing machines, not including |  |  |  |  |  |
| 1. Number of bathrooms |  |  |  |  |  |
| 1. DVD player, including any device that can read DVDs and not considering vehicle DVDs |  |  |  |  |  |
| 1. Number of fridges |  |  |  |  |  |
| 1. Number of freezers unconnected to or part of a duplex fridge |  |  |  |  |  |
| 1. Number of computers, considering desktops, laptops, notebooks, netbooks and not considering tables, palms or smartphones |  |  |  |  |  |
| 1. Number of dishwashers |  |  |  |  |  |
| 1. Number of microwaves |  |  |  |  |  |
| 1. Number of motorcycles, not considering those used for work |  |  |  |  |  |
| 1. Number of washer-dryers |  |  |  |  |  |

| **Where does the running water come from?** |
| --- |
| ❑ 1. General water distribution system  ❑ 2. Well or spring  ❑ 3. Other means |

| 1. **Considering your stretch of street, you would say it is:** |
| --- |
| ❑ 1. Asphalted/Paved  ❑ 2. Dirt/gravel |

What is the education level of the **head of the family?** Take the head of the family as the person who contributes with most of the household income.

|  | **Current terminology** |
| --- | --- |
| ❑ | Iliterate/ Unfinished middle School I |
| ❑ | Middle School I/ Unfinished middle school II |
| ❑ | Middle school/ Unfinished high school |
| ❑ | High school/Unfinished college degree |
