# Supplementary material for: Health-related quality of life in Brazilian patients with cutaneous leishmaniasis using EQ-5D
Source: PLoS One. 2025 May 23;20(5):e0324788. doi: 10.1371/journal.pone.0324788 (PMC12101690; doi:10.1371/journal.pone.0324788)
Supplement: Attachment — Submitted filename: Response to Reviewers_26.3.25.docx [file pone.0324788.s005.docx]

April 3, 2025

Dear Vinícius Silva Belo

PLOS ONE

We would like to thank the reviewers for their careful review of the manuscript *"Health-related quality of life in patients with cutaneous leishmaniasis in Brazil using EQ-5D*" (PONE-D-24-57829), now with the changed title “*Health-related quality of life in Brazilian patients with cutaneous leishmaniasis using EQ-5D”*. This opportunity to improve our work makes it even more robust and useful to the scientific community - our sole goal.

We did our best to improve the text accordingly. The modifications implemented in this revised version are summarized below together with specific answers to the questions placed by the reviewers.

We reinforced that the funders had no role in study design, data collection and analysis, decision to publish, or preparation of the manuscript.

We hope the reviewers find our responses to their comments satisfactory. Please let us know if any further improvements to the manuscript are needed.

Sincerely yours,

Corresponding author:

Sarah Nascimento Silva

Instituto René Rachou - Fundação Oswaldo Cruz – Fiocruz Minas

Avenida Augusto de Lima, 1715

30190-001, Belo Horizonte, MG, Brazil

Phone/fax:	+ 55 31 3349-7782 - E-mail: [sarah.nascimento@fiocruz.br](mailto:sarah.nascimento@fiocruz.br)

**Answer Letter to Reviewers**

| **Local** | **Reviewer comment** | **Author response** | **Alterations in manuscript** |
| --- | --- | --- | --- |
|  | **Reviewer #1** |  |  |
| Overall impression | The authors evaluated the performance of the CLIQ using the EQ-5D-3L as a reference. The manuscript technically sound, the data supported the conclusions and is presented in an intelligible fashion and written in standard English. However, I have some suggestions. | We appreciate this feedback and the chance to improve our manuscript.  We carefully read your recommendations and made changes accordingly. | All changes in the manuscript are highlighted. |
| Abstract  Line 27 | Review the phrase: "in patients with CL in patients." There is a repetition of terms ("in patients") | The sentence was revised. | Abstract, lines 44 and 45 |
| Abstract  Objective: | I suggest removing "in patients attending a Brazilian reference center, using the EQ-5D-3L/VAS questionnaire to generate utility measures for this disease," as this information is better suited for the "Methods" section. | The suggestion was accepted. | Abstract, lines 45. |
| Abstract  Methods | The study design is described as cross-sectional; however, the methodology presents the same comparison variables within the same population at different moments (before the LC diagnosis and after the onset of the first symptoms), which would characterize it as longitudinal. | Thank you for raising this perspective.  The suggestion was accepted.  The study design has been changed to “retrospective longitudinal”. | Abstract, line 45. |
| Abstract  Results | The sentence "The mean utility score before the onset of CL symptoms was 0.858. A comparison of the responses related to health status before and after the illness revealed significant losses (p<0.05) in all dimensions of the EQ-5D, especially those related to pain, malaise, and usual activities, which were reduced to 50% and 27%, respectively" seems paradoxical. The way it is written suggests a reduction in pain, malaise, and activities. I suggest rewording this sentence. | Thank you for this comment. The suggestion was accepted. | Abstract, lines 52-55 |
| Introduction | I suggest explicitly stating the objective in the introduction | Thank you for this comment. We have included the objective explicitly, as suggested  Added the word “aim” to the sentence (end of Introduction section) | Introduction, line 118 |
| Methods  Study design | Considering that the same individuals were evaluated with the same variables at different time points (before the onset of symptoms and during the active phase of CL), I suggest revising the study design to be described as longitudinal. | The suggestion was accepted. The study design has been changed to “retrospective longitudinal” and the changes in the manuscript are highlighted. | Methods  First part of Subsection “Study design and data collection”, line 173. |
| Methods  Study design | I suggest clarifying the variable used to assess health status before and during the active phase of CL. For example: How was the timeframe determined to define the period before symptom onset? (One week before? One month before? One year before?). Regarding the active disease phase, it is crucial to specify that it was 30 days after symptom onset. | Thank you for this suggestion. It was accepted.  We have added statements clarifying the timeframe for responding to each health status. | Methods  Subsection “Study design and data collection”, lines 187-191 |
| Methods  Study design | The age range of participants varied from adolescence (12 years and older) to elderly individuals (over 74 years old). It would be essential to explain the rationale for selecting this age range because quality of life is sensitive to age. For example, mobility or self-care (as evaluated in the questionnaire) before symptom onset and during the active disease phase may not be comparable between a 12-year-old individual and someone over 74. | The study aimed to capture the broad impact of cutaneous leishmaniasis (CL) across different life stages, as the disease affects individuals of all ages. Including participants 12 years and older was essential to represent the full spectrum of patients affected by CL in endemic areas and to align with the eligibility criteria for treatment at the reference center.  We recognize that certain dimensions of the EQ-5D-3L questionnaire, such as mobility and self-care, may have different baseline expectations for adolescents compared to elderly individuals. To account for this, we performed stratified analyses by age groups, as now presented in Table 1, allowing for a more nuanced interpretation of the results. Additionally, statistical comparisons between these groups help contextualize potential differences in QoL changes before and during active CL. We have included a discussion on this matter in the revised manuscript to acknowledge these potential limitations and ensure transparency in our interpretation of the findings. | Methods: First part of Subsection “Study design and data collection”, lines 176-180  Table 1 has been updated.  Discussion section, lines 530 – 536. |
| Methods  Study design | It would be essential to clarify whether an analysis was conducted to assess quality of life in association with socioeconomic, sociodemographic, and clinical variables, such as age and comorbidities. | Thanks for your comment. We have revised Table 1 to explicitly present these associations. The updated table, which replaces the previous version, now includes utility scores for both pre-disease and active disease phases, along with their respective p-values. This revision directly addresses the relationship between HRQoL and variables such as sex, age, socioeconomic status, education level, comorbidities, and other clinical factors. By incorporating this new table, we provide a more transparent and structured presentation of the impact of these variables on HRQoL. The statistical significance of differences across subgroups is now explicitly stated, ensuring a clear interpretation of how socioeconomic, sociodemographic, and clinical factors influence HRQoL in patients with cutaneous leishmaniasis. We hope this modification adequately addresses your concern. | Table 1 has been updated.  The results for this association have been moved so that they appear at the end of the second paragraph of the Results section (lines 353 – 357) |
| Methods  Study design | I suggest further clarifying the inclusion criteria. For example, were individuals with a prior diagnosis of depression and/or anxiety included in the study? | Thank you for this comment. All patients diagnosed with cutaneous leishmaniasis (CL) between October 1st, 2020, and April 30th, 2023, who were 12 years or older, were eligible for inclusion, without specific exclusion criteria. Therefore, individuals with a prior diagnosis of depression and/or anxiety were not excluded from the study. We have revised the manuscript to explicitly state the inclusion criteria and clarify that no patients were excluded based on prior mental health diagnoses. | Methods section, first part of Subsection “Study design and data collection”, lines 179-180. |
| Results | I believe it would be more objective to concisely describe the convenience sample relative to the total eligible population. | Thank you for your valuable suggestion. We have explicitly stated that we enrolled a convenience sample consecutively recruited and have reworded the relevant sentence to improve clarity. The revised text now reads: "A total of 152 patients were invited to participate, representing 47% (152/321) of the total eligible population during the study period. Of these, 143 individuals (93% of those invited) agreed to participate." | Methods section, 1st paragraph, line 175.  Results section, 1st paragraph, lines 316-320. |
| Results | The statement "The VAS results support the magnitude of this loss, with the average score reported by patients before CL being 87 and decreasing to 70 during active CL, representing a loss of 19.5%" needs a clearer statistical description to demonstrate that this score reduction on the VAS was significant and independently associated with the CL diagnosis. (It is stated that more than 50% of participants had comorbidities such as diabetes and hypertension, some of whom were untreated, which could be a factor affecting vision.) | We appreciate the opportunity to clarify and strengthen this aspect of our analysis. To address this, we have now explicitly stated that the difference in VAS scores before and during active CL was statistically significant (p < 0.001). | Results section, 3rd paragraph, line 368. |
| Results | I suggest avoiding repetition of information from the table in the text, as it becomes redundant. Instead, keep only complementary details in the text. | Thank you for this comment. We have revised the first and second paragraphs of the Results section to reduce the numerical data in the text. | Results section, 1^st^ and 2^nd^ paragraphs, lines 316 – 357. |
| Results | I recommend completing the table titles so that they can be interpreted and understood independently of the text. For example, missing details include location and study period. | Thank you for this suggestion. We've included more details in the table title. | The title of Table 1 has been updated, lines 322 – 324. |
| Results | The age groups in the results table were categorized in a non-standard manner. I suggest explaining this categorization in the Methods section or providing a reference to justify it. I reinforce that the broad age range may impact quality of life differently among individuals with CL. | The age groups were categorized based on epidemiological relevance and sample distribution, ensuring a balanced representation of participants across different life stages. While no predefined standard was strictly followed, this categorization allowed for meaningful comparisons within the study population. To address your concern, we have now clarified this rationale in the Methods section. We acknowledge that the broad age range may influence quality of life differently among individuals with CL. However, as noted in our analysis, there were no significant differences in utility values across age categories, suggesting a consistent impact of CL on HRQoL. We have reinforced this discussion in the manuscript to ensure transparency in the interpretation of our findings. | Methods section, Data collection instruments  subsection last paragraph, lines 282 – 285. |
| Results | The figures are not clear; I suggest improving their resolution for better readability. | Thank you for your valuable feedback. We have made every effort to improve the quality of the figures using the NAAS tool, as recommended by the journal's editors, and have achieved the best possible resolution within the tool's capabilities. | Fig 1 and Fig 2 |
|  | **Reviewer #2** |  |  |
| Overall impression | Its interesting manuscript about the "Health-related quality of life in patients with cutaneous leishmaniasis in Brazil using EQ-5D".  The manuscript presents a well-structured study on the impact of cutaneous leishmaniasis (CL) on health-related quality of life (HRQoL) using the EQ-5D-3L instrument in a Brazilian cohort. The study addresses an important gap in the literature by quantitatively assessing the disease burden using standardized measures. The methodology is sound, and the statistical analyses are appropriate. However, there are some areas that require improvement in structure, clarity, and interpretation of results. | Thank you for your valuable evaluation. We carefully read your recommendations and made changes accordingly. | All changes in the manuscript are highlighted. |
| Title and Abstract | Title: The title is clear and informative but could be slightly refined for conciseness. Suggested revision: "Health-Related Quality of Life in Brazilian Patients with Cutaneous Leishmaniasis Using EQ-5D-3L". | The suggestion was accepted. | The title was change to: “Health-related quality of life in Brazilian patients with cutaneous leishmaniasis using EQ-5D-3L" |
| Abstract | The abstract is well-structured but contains minor grammatical errors and awkward phrasing.  The sentence "The aim of the present study was quantitatively assess the health-related quality of life..." should be rewritten as: "The aim of this study was to quantitatively assess the health-related quality of life..." | Thank you for this comment. The suggestion was accepted, and the sentence has been rewritten. | Abstract, lines 44-45 |
| Abstract | The conclusion could be more impactful by emphasizing the broader implications for public health and policy. | Thank you for your valuable feedback. We have revised the conclusion to emphasize the broader implications for public health and policy, highlighting the potential impact of our findings on healthcare strategies and the allocation of resources for the management of dermatological conditions. | Abstract, lines 56-80 |
| Introduction | The introduction provides a strong rationale for the study, outlining the burden of CL and the importance of HRQoL assessment. | Thank you for this comment. | None. |
| Introduction | The references are relevant, but the discussion of previous studies could be slightly condensed to improve readability. | Thank you for this comment. We've reduced the sentence as suggested. | Introduction, lines 101-108 |
| Abstract introduction | The sentence "However, no studies were found that quantitatively defined the health utility of CL." might be misleading because studies have assessed QoL in CL patients using different instruments. Instead, it should specify that no utility-based HRQoL assessments had been conducted. | Thank you for this comment. We have changed the sentence as suggested | Abstract, lines 43-44 |
| Methods | The study design and statistical approach are well-described. | Thank you for this comment. | None. |
| Methods | The retrospective assessment of pre-disease HRQoL introduces recall bias, which is acknowledged, but additional justification on why this approach was necessary could strengthen the discussion. | Thank you for this suggestion. We have added statements justifying the retrospective assessment. | Methods section, final part of Subsection “Study design and data collection”, lines 190-191. |
| Methods | The use of EQ-5D-3L is appropriate, but a brief mention of why this tool was preferred over disease-specific QoL instruments (e.g., Dermatology Life Quality Index) would be beneficial. | Thanks for the comment. As previously mentioned in the Introduction, we highlighted the advantage of using generic instruments to facilitate comparisons across different interventions and economic evaluations. To address your suggestion, we have now made this reasoning more explicit and expanded the explanation to reinforce why the EQ-5D-3L was selected | Introduction section, last paragraph, lines 119-122. |
| Methods | The sample size justification is missing—was a power calculation conducted? | Thank you for your comment. No formal power calculation was conducted prior to data collection, as this study was based on a convenience sample of patients diagnosed with CL at a Brazilian reference center within the study period. Our goal was to include as many eligible patients as possible to ensure a representative assessment of HRQoL in this population. However, we acknowledge the importance of addressing sample size considerations. To strengthen the manuscript, we have explicitly stated this in the Methods section, clarifying that the sample was defined based on feasibility and patient availability rather than a priori statistical power estimation. | Methods section, 1st paragraph of Subsection “Study design and data collection”, lines 176-179. |
| Results | The results are clearly presented with appropriate statistical analyses. | Thank you for this comment. | None. |
| Results | Figures and tables are well-organized but should be cross-referenced more explicitly in the text. | Thank you for your comment.  We double checked that all tables have been properly referenced in the results section. | The figures and tables are already referenced in bold throughout the text. |
| Results | The finding that pain/discomfort and usual activities were the most affected dimensions is expected but could be better contextualized with references to similar conditions (e.g., post-herpetic neuralgia, chronic skin conditions). | Thank you for this comment. The contextualization of the effects in each dimension was done in the discussion section, comparing with other studies for CL.  However, to address your recommendation, we have further contextualized these findings by incorporating references to studies on other dermatological conditions. | Discussion section, 2^nd^ paragraph, lines 438-449. |
| Results | There is no discussion of potential sex-based differences despite reporting a significant difference in baseline HRQoL scores between men and women. | Thank you for your insightful comment. We have incorporated a discussion of this difference in the revised manuscript. | Discussion section, 7^th^ paragraph, lines 498 – 509. |
| Discussion | The discussion effectively summarizes key findings and places them in context. | Thank you for this comment | None. |
| Discussion | The authors compare their utility scores with those from studies on other diseases (e.g., HIV, herpes zoster), which is useful but should be expanded to explain why these comparisons are relevant. | Thank you for your valuable feedback. We have revised the Discussion section to include comparisons only with studies on dermatological diseases, such as CL. | Discussion section, 2^nd^ paragraph, lines 438-449. |
| Discussion | Some speculative statements require more evidence, e.g., "These findings underscore the severity of the impact of CL on QoL, which is comparable to that of other chronic and debilitating diseases."—This should be supported by additional references or phrased more cautiously. | Thank you for this comment. The sentence has been restructured more cautiously | Discussion section, 2^nd^ paragraph, lines 448-449. |
| Discussion | The limitations section is comprehensive, but it does not address potential selection bias—since patients were recruited from a referral center, they may represent more severe cases of CL. | Thank you for your suggestion. We've added this potential source of bias to the discussion | Discussion section (11^th^ paragraph), lines 550-552. |
| Conclusion | The conclusion aligns with the study’s findings but could be more impactful by emphasizing the implications for clinical care and policymaking. | Thank you for your suggestion. The revised conclusion now highlights how the findings contribute to a better understanding of the burden of CL on HRQoL and underscores the importance of incorporating utility-based measures in economic evaluations and health policy decision-making. | Conclusion, lines 563 – 584. |
| Conclusion | The sentence "To maximize the value of these studies, it is crucial to continue enhancing assessment instruments..." is vague—what specific improvements are suggested? | We have updated the conclusion in the revised manuscript to provide a clearer and more specific perspective on how HRQoL assessments can be enhanced in future studies. | Conclusion, lines 563 –584 |
|  | **Reviewer #3** |  |  |
| Overall impression | The manuscript provides valuable insights into the health-related quality of life (HRQoL) in cutaneous leishmaniasis (CL) patients using the EQ-5D-3L/VAS questionnaire. The study is well-designed, and the data appropriately support the conclusions. However, some minor revisions are recommended: | We sincerely appreciate the reviewer's valuable feedback and the opportunity to improve our manuscript. | All changes in the manuscript are highlighted. |
| Clarity & Readability | Some sentences need rewriting. For example, “The aim of the present study was quantitatively assess…” should be revised to “The aim of this study was to quantitatively assess…”. Consider reviewing sentence structures for clarity. | Thank you for this comment. We have changed the sentence as suggested. | Abstract, lines 44-45. |
| Terminology | Replace “transversal study” with “cross-sectional study” for better clarity, as the latter is the standard term in English-language research. | Thank you for your comment. We have indeed changed the name of the study design for clarity. | The study design has been changed to “retrospective longitudinal”.  Abstract, line 46.  Methods, line 173. |
